# Primary healthcare providers’ knowledge, attitudes, and practices regarding cancer screening recommendation and referral in Georgia, 2023

**DOI:** 10.1080/13814788.2025.2582292

**Published:** 2025-11-24

**Authors:** Marina Topuridze, Ana Kareli, Givi Javashvili, Nino Kiknadze, Nino Shiukashvili, Teona Todua, Tamar Melikidze, Ketevan Khetsuriani, Davit Otiashvili

**Affiliations:** a Health Promotion and Communication Division, National Center for Disease Control and Public Health, Tbilisi, Georgia; b Scientific-Research and PhD Department, Petre Shotadze Tbilisi Medical Academy, Tbilisi, Georgia; c Family Medicine Department, Tbilisi State Medical University, Tbilisi, Georgia; d Basic Sciences Department, Ken Walker International University, Tbilisi, Georgia; e Faculty of Natural Sciences and Medicine, Ilia State University, Tbilisi, Georgia

**Keywords:** Cancer screening, primary healthcare, referral practices, healthcare providers, policy reform, Georgia (country)

## Abstract

**Introduction:**

Cancer screening uptake in Georgia is only 13%. We aimed to assess cancer screening recommendations and referral practices among primary health care providers (PHC) and factors associated with these practices.

**Methods:**

We conducted a cross-sectional survey of 2,958 doctors and nurses in PHC in November 2023, using descriptive, bivariate, and multivariate analyses to assess the association between provider characteristics, system-level factors, and practices in recommending and referring for cancer screening.

**Results:**

Respondents were predominantly female (95%), mean age of 57.5 years, family doctors (56%), with >20 years’ work experience (60%) and rural practice (67%). Almost all (98%) reported actively recommending and referring patients for cancer screening; however, 64.7% did so for only 1–5 patients per week, while 12% reported none. Predictors of higher recommendation and referral rates (≥6 patients per week) included being a family doctor (AOR 1.36; 95% CI: 1.11–1.67; *p* = 0.003), working in the capital city – Tbilisi (AOR 1.36; 95% CI: 1.14–1.95; *p* = 0.003), receiving comprehensive cancer screening training (AOR 1.49; 95% CI: 1.04–1.68; *p* = 0.024), reporting adequate infrastructure (AOR 1.39; 95% CI: 1.07–1.81; *p* = 0.015), reporting proper public promotion (AOR 1.41; 95% CI: 1.12–1.78; *p* = 0.003), and perceiving screening as part of their role (AOR 1.87; 95% CI: 1.52–2.30; *p* < 0.001).

**Conclusions:**

Despite strong belief in cancer screening, recommendation and referral rates remain low, underscoring the urgent need for policy reforms to enhance education, raise awareness, and improve infrastructure for effective cancer screening initiatives.

## Introduction

Cancer is a major public health concern in Georgia, with mortality-to-incidence ratios and survival rates significantly worse than those in other WHO European Region countries [1]. In 2022, Georgia reported 13,689 new cancer cases, reflecting an age-standardised incidence rate of 197.5 per 100,000 population, with 39% diagnosed at advanced stages (III and IV), aligning with trends in neighbouring countries [2,3]. The age-standardised cancer-related mortality rate was 111.7 per 100,000, with 8,459 cancer-related deaths reported [2].

Cancer screening reduces morbidity and mortality by enabling early detection and treatment, lowering costs and improving survival [4]. This is especially important in low- and middle-income countries, where late-stage diagnosis is common [5]. The WHO recommends at least 70% coverage in the eligible population to maximise impact [6].

In Georgia, free screening programs for breast, cervical colorectal cancers have been available national wide since 2011 [7]. However, uptake remains extremely low, particularly outside Tbilisi, mirroring trends in many LMICs [7,8]. In 2023, the participation rate for breast cancer screening was only 16%, 15% for cervical cancer, and 3% for colorectal cancer.

Globally, studies have shown that provider endorsement is one of the strongest predictors of patient engagement in screening programs [9–11]. In 2020, Georgia adopted an organised cancer screening model, expanding PHC providers’ roles to ensure at least 25% of eligible beneficiaries in their municipalities participate annually. Since 2023, their responsibilities have included enrolling patients, scheduling appointments via an electronic system, and conducting educational outreach to raise awareness [12].

A critical gap exists in understanding PHC providers’ cancer screening recommendations and referral practices, as well as the factors influencing them. Limited and outdated research suggests they often lack knowledge of screening guidelines [13] and rarely recommend screenings [14].

This study aims to evaluate the cancer screening recommendation and referral practices of 2,985 PHC providers in Georgia. It examines variations by specialty, demographics and location while identifying key barriers and enablers affecting these practices. By addressing these gaps, the findings aim to inform strategies to enhance PHC engagement and improve cancer screening uptake in Georgia and similar LMICs.

## Methods

### Study design

This cross-sectional survey, conducted in November 2023, assessed PHC providers’ practices in recommending and referring patients for cancer screening, as well as the factors influencing these practices.

### Study population

The survey targeted PHC providers from urban and rural settings across Georgia who was enrolled in a state-funded continuous medical education (CME) program on cancer screening. Of 3,249 invited trainees, 3,097 (95.3%) agreed to participate. After initial agreement, 112 participants were excluded from the final analysis due to incomplete questionnaires or missing essential outcome data, resulting in a final sample of 2,985 family doctors and nurses, who, under national policy, are directly responsible for patient communication and referrals to cancer screening programs.

### Setting

Georgia’s primary healthcare (PHC) workforce comprises approximately 5,000 staff, including family doctors, nurses, and subspecialists, 80% of whom are women aged 45–60. The PHC system operates under distinct urban and rural models: rural healthcare is managed by the state-owned Georgian Medical Holding, while urban practices are privately owned and contract with the National Health Agency under the Universal Healthcare Program. The system collectively serves around 3 million people, with many providers exceeding the recommended patient limit of 2,500. In 2019, 6% of rural doctors and 28% of urban doctors managed more than 2,000 patients, highlighting significant strain on the system [15]. Nurses, working alongside family doctors, share responsibility for patient education, recommendations, and facilitating screening appointments, often via an electronic booking system; direct patient self-referral to screening programs is also possible.

### Procedures

The Association of Family Doctors of Georgia (AFDG) administered the survey. Participants completed a structured, self-administered questionnaire before their training sessions, either on paper or tablet. Responses were collected in one round, entered into a database, and checked for consistency.

### Measurements

The survey, adapted from the International Agency for Research on Cancer (IARC) tool (Supplementary File 1), included 42 questions across multiple domains:

- **Provider and Facility Characteristics**: Gender, age, specialty, work experience, and PHC setting type.
- **System-Level Factors**: Availability of guidelines, training programs, infrastructure, coordination, public promotion, recall systems, and electronic tools.
- **Provider-Level Factors**: Knowledge, motivation, capacity, and perceptions of cancer screening.

The primary outcome was the number of patients recommended and referred weekly, initially categorised into five groups (0, 1–5, 6–10, 11–20, and >20). This variable included both direct and indirect interactions, such as informing patients about services or assisting with scheduling appointments via the online system, through phone calls or in-person discussions during routine visits. We consolidated these categories into two groups (0–5 and ≥6) based on a Chi-Square Test of Independence, which indicated no significant differences between adjacent categories. Additionally, sensitivity analyses using alternative cut-offs (≥4, ≥8, and ≥10) were conducted, and consistent results were found across these thresholds.

Knowledge was assessed separately for each cancer type and combined into a composite scale (range: 0–7). Cronbach’s alpha confirmed internal consistency (≥0.7) [16]. Motivation, opportunity, and perceptions were measured on a 5-point Likert scale [17] and were collapsed into dichotomous categories (‘Agree’ vs. ‘Other’) for analysis.

### Statistical methods

Data analysis was conducted using SPSS (v26.0). Descriptive statistics were used to summarise participant demographics and responses. Chi-square tests examined associations between various factors and referral practices, stratified by urban/rural settings and provider roles (nurses vs. doctors). Multivariate analysis was performed to adjust for confounders and identify key system- and provider-level predictors of screening recommendation and referral practices.

Covariate selection for the multivariable models was informed by literature on determinants of screening among PHC providers, including provider characteristics (specialty, experience, gender), practice setting (urban/rural), and system/provider factors (training, infrastructure, coordination, perceived responsibility). These variables have been linked to preventive care engagement and screening uptake [9–12,14,18].

### Ethics

The appropriate institutional research ethics committee approved the study. Participation was voluntary, and informed consent (written or electronic) was obtained from all participants. No conflicts of interest were reported.

## Results

Of the 2,985 participants, 95% were female, with a mean age of 57.5 years. Most were family doctors (56%) with over 20 years of PHC experience (60%; mean: 25 years). Two-thirds (67%) worked in rural settings, with the largest group residing in the Imereti region (18%), followed by Tbilisi, Samegrelo-Zemo Svaneti, and Kakheti (12% each) (Table 1).

**Table 1.** Healthcare provider and facility characteristics stratified by specialty, Georgia, 2023.

Variables/Provider Characteristics	Total		Family doctors		Nurses		
	(*N* = 2985)	%	(*N* = 1665)	%	(*N* = 1320)	%	*p*-value
**Specialty**							
Family Doctor	1665	55.78	–	–			
Nurse	1320	44.22	–	–			
Work experience in PHC* (Mean, SD)**	**25.6 (SD 12.9)**		25.7 (SD 12.3)		25.5 (SD 13.7)		0.785
**Gender**							
Male	145	4.86	142	8.5	3	0.2	<0.001
Female	2840	95.14	1523	91.5	1317	99.8	
**Age** (Mean, SD)	57.48 (SD 10.1)		58.8 (SD 9.4)		55.9 (SD 10.7)		<0.001
**PHC facility location type**							
Rural	**2012**	**67.4**	1052	63.2	960	72.7	<0.001
Urban	**973**	**32.6**	613	36.8	360	27.3	
**PHC facility location Region**							
Imereti	533	17.9	287	17.2	246	18.6	<0.001
Tbilisi	371	12.4	257	15.4	114	8.6	
Samegrelo–Zemo Svaneti	366	12.3	191	11.5	175	13.3	
Kakheti	368	12.3	210	12.6	158	12.0	
Adjara	293	9.8	180	10.8	113	8.6	
Kvemo Kartli	256	8.6	129	7.7	127	9.6	
Shida Kartli	198	6.6	144	8.6	54	4.1	
Samtskhe–Javakheti	164	5.5	70	4.2	94	7.1	
Guria	149	5.0	75	4.5	74	5.6	
Racha–Lechkhumi and Kvemo Svaneli	146	4.9	51	3.1	95	7.2	
Mtskheta–Mtianeti	141	4.7	71	4.3	70	5.3	

The majority of participants (98%) reported actively recommending cancer screening. A total of 21.2% reported recommending and referring screening to 6 or more patients per week, with 14.3% doing so for 6–10 patients, 3.2% for 11–15 patients, 1.5% for 16–20 patients, and 2.2% for more than 20 patients. Most participants recommended and referred screening to 1–5 patients per week (64.8%), while 14.1% reported providing no recommendations or referrals (Table 2).

**Table 2.** Cancer screening recommendation and referral practices of primary healthcare providers and system level factors stratified by specialty, Georgia, 2023.

Variables/Provider Characteristics	Total		Family doctors		Nurses		
	(*N* = 2985)	%	(*N* = 1665)	%	(*N* = 1320)	%	*p*-value
Practice related to facilitating cancer screening							
Actively recommend cancer screening (Yes)	2936	98.4	1574	94.5	1264	96.1	0.242
Number of patients referred for cancer screening							
0	357	12.0	210	58.8	147	41.2	0.016
1–5	1933	64.7	1039	53.8	894	46.2	
6–10	426	14.3	253	59.4	173	40.6	
11–15	95	3.2	62	65.3	33	34.7	
16–20	45	1.5	31	68.9	14	31.1	
More than 20	66	2.2	34	51.5	32	48.5	
Healthcare system-level factors to cancer screening							
Cancer screening program is defined by a policy framework (Yes)	2693	90.2	1530	91.9	1163	88.1	<0.001
Protocol/guideline for cancer screening (Yes)	2562	85.8	1449	87.0	1113	84.3	0.035
Trained on cancer screening including how to advise people to get screened, benefits and risks (Yes)	2250	75.6	1276	76.8	974	74.0	0.076
System in place for identifying target population (Yes)	1737	58.2	1046	62.8	619	37.2	<0.001
System in place for inviting eligible individuals for screening (Yes)	1159	38.8	660	39.6	499	37.8	0.307
System in place for notifying the results and informing about follow-up (Yes)	1184	39.7	702	42.2	482	36.5	0.002
System in place for sending recall notice to the non-compliant individuals (Yes)	696	23.0	427	25.6	269	20.4	<0.001
Evaluation of performance of the programme which is published and accessible for providers (Yes)	446	14.9	243	14.6	203	15.4	0.551
Sufficient number of providers to ensure screening programme (Yes)	385	12.9	235	14.1	150	11.4	0.026
High staff turnover, which prevents proper continuity of the programme (Yes)	142	4.8	78	4.7	64	4.8	0.835
Adequate infrastructure for screening (Yes)	637	21.3	397	23.8	240	18.2	<0.001
Adequate infrastructure for further management (Yes)	363	12.2	245	14.7	118	8.9	<0.001
Long waiting lists for screening (Yes)	144	4.8	73	4.4	71	5.4	0.208
Long waiting lists for further management in case of screen positive result (Yes)	179	6.0	95	5.7	84	6.4	0.452
Adequate coordination between different healthcare levels (Yes)	377	12.6	236	14.2	141	10.7	0.004
Proper public promotion of the screening programme (Yes)	723	24.2	434	26.1	289	21.9	0.008
Cancer screening visit registration electronic system for patients (Yes)	792	26.5	484	29.1	308	23.3	<0.001

Most respondents (90%) reported that cancer-screening programs were guided by national policy frameworks, and 86% had access to protocols or guidelines. While 75% had received training, a minority perceived adequate staffing (13%), screening and management infrastructure (21% and 12%, respectively), and coordination across medical service levels (13%) or public promotion (24%). Delays in accessing screening services or managing positive cases were reported by fewer than 6% (Table 2).

Knowledge about target age groups and screening frequency varied widely (correct responses ranged from 39% to 75% by cancer type). The average knowledge score was 3.3 (SD = 1.6) out of 7. The majority of PHC personnel (99%) believed cancer screening effectively reduces morbidity and mortality. While 70% considered themselves well-informed about screening procedures, 69% perceived their role as important in increasing screening uptake (Table 3).

**Table 3.** Cancer screening related knowledge and perceptions of primary healthcare providers stratified by specialty, Georgia, 2023.

Variables/Provider Characteristics	Total		Family doctors		Nurses		
	(*N* = 2985)	%	(*N* = 1665)	%	(*N* = 1320)	%	*p*-value
Knowledge							
Knows about the national cervical cancer screening target age	2236	74.9	1255	75.4	981	74.3	0.508
Knows about the national breast cancer screening target age	1792	60.0	1022	61.4	770	58.3	0.107
Knows about the national colorectal cancer screening target age	1548	51.9	910	54.7	638	48.3	<0.001
Knows about the cervical cancer screening frequency	1577	52.8	851	51.1	557	42.2	<0.001
Knows about the national breast cancer screening frequency	1763	59.1	1039	62.4	724	54.8	<0.001
Knows about the national colorectal cancer screening frequency	1165	39.1	646	38.8	519	39.3	0.773
Knowledge about cancer screening program. Scale (Mean, SD)	3.3 (SD 1.6)		3.4 (SD 1.55)		3.2 (SD 1.57)		0.984
Perceptions							
Believe that cancer screening is effective in decreasing cancer morbidity (Yes)	2955	98.9	1657	99.5	1297	98.3	<0.001
Believe that cancer screening is effective in decreasing cancer mortality (Yes)	2960	99.2	1657	99.2	1308	99.1	0.921
Motivated to recommend cancer screening to patients							
Strongly agree	1047	35.1	700	42.1	347	26.3	0.334
Somewhat agree	1889	63.3	941	56.5	948	71.8	
Other*	49	1.6	24	1.4	25	1.9	
Well informed about cancer screening procedures							
Strongly Agree	584	19.6	380	22.8	204	15.5	<0.001
Somewhat agree	2092	70.1	1122	67.5	970	73.5	
Other	309	10.3	163	9.7	146	11.0	
Role of primary healthcare provider in cancer screening is important							
Strongly agree	789	26.4	517	31.1	272	20.6	<0.001
Somewhat agree	2068	69.3	1094	65.7	974	73.8	
Other	128	4.3	54	3.2	74	5.6	
Have enough time to discuss cancer screening program to the patients							
Strongly agree	401	13.4	227	13.6	174	13.2	0.719
Somewhat agree	2132	71.4	1120	67.3	1012	76.7	
Other	452	15.2	318	19.1	134	10.1	
Cancer screening is higher priority than other healthcare issues							
Strongly agree	598	20.0	356	21.4	242	18.3	0.039
Somewhat agree	2160	72.4	1153	69.2	1007	76.3	
Other	227	7.6	156	9.4	71	5.4	
Part of my job is to help people make an informed decision							
Strongly agree	754	26.1	450	27.9	304	23.8	0.011
Somewhat agree	2042	70.6	1110	68.9	932	72.9	
Other	94	3.3	52	3.2	42	3.3	
Confident that can help people make an informed decision							
Strongly agree	622	21.6	368	22.9	254	19.9	0.747
Somewhat agree	2137	74.0	1166	72.4	972	76.2	
Other	127	4.4	76	4.7	50	3.9	
Having necessary knowledge to help people make an informed screening decision							
Strongly agree	481	16.7	277	17.3	204	15.9	0.430
Somewhat agree	2117	73.4	1165	72.6	953	74.5	
Other	286	9.9	163	10.1	122	9.6	

*Other – combines responses: Strongly disagree, disagree and do not know.

### Stratified analysis

Significant differences (*p* ≤ 0.05) were observed across provider roles and locations:

**Nurses vs. Family Doctors**: Nurses were less likely to refer ≥6 patients weekly, demonstrate awareness of screening policies (88% vs. 92%), of protocols and guidelines (84% vs. 87%) or correctly identify target groups (Table 2). They were also less likely to strongly agree that they are well informed about cancer screening procedures (16% versus 23%) and that their role in cancer screening was important (21% vs. 31%) (Table 3). Additionally, nurses were less likely to perceive adequate screening infrastructure (18% vs. 24%) or coordination between healthcare levels (11% vs. 14%) and reported lower awareness of the electronic system for cancer screening registration (22% vs. 26%). They were less likely to believe in the effectiveness of cancer screening in reducing cancer morbidity (98% vs. 100%) (Table 3).

**Rural vs. Urban Providers:** Rural providers were more aware of policy frameworks (92% vs. 88%) and screening protocols (88% vs. 82%) but faced greater challenges with follow-up systems (37% vs. 41%) and patient recalls (20% vs. 25%). They were less knowledgeable about cervical cancer screening frequency (46% vs. 50%) and less likely to strongly agree they were well-informed about screening procedures (18% vs. 22%) or that their role in cancer screening is important (25% vs. 30%). Given data can be found in the Supplementary Tables 1–3.

### Multivariate analysis

Independent statistically significant (*p* ≤ 0.05) predictors of recommending and referring ≥6 patients per week were: (1) being family doctor (OR = 1.36 95% CI: 1.11–1.67 *p* = 0.003); (2) working in Tbilisi (OR = 1.36 95% CI: 1.14–1.95 *p* = 0.003) (3) receiving comprehensive training on cancer screening (including how to advise people to get screened, as well as benefits and risks) (OR = 1.49 95% CI: 1.04–1.68, *p* = 0.024); (4) perceiving adequate infrastructure for screening (OR = 1.39, 95% CI: 1.07–1.81, *p* = 0.015); (5) perceiving that there is a proper public promotion of the screening program among the population (OR = 1.41, 95% CI: 1.12–1.78, *p* = 0.003) and (6) Viewing screening as part of their role (OR = 1.87, 95% CI: 1.52–2.30, *p* < 0.001) (Table 4).

**Table 4.** Multivariate analysis results of the factors associated with cancer screening recommendation behaviour among primary healthcare providers, Georgia, 2023.

Variables	Provides recommendation and referral to cancer screening to 5+ patients a week*		
	Adjusted OR	95 % CI	*p*-value
**Specialty**			
Family doctor	1.36	1.10-1.67	0.003
Nurse	Ref		
**Region of Work**			
Tbilisi	1.49	1.14–1.95	0.003
Other Regions	Ref		
**Received training on cancer screening**			
Yes	1.32	1.04–1.68	0.024
No	Ref		
**There is an adequate infrastructure for screening**			
Yes	1.39	1.07–1.81	0.015
No	Ref		
**There is a proper public promotion of the screening programme**			
Yes	1.41	1.12–1.78	0.003
No	Ref		
**Part of my job is to help people make an informed decision to be screened**			
Strongly Agree	1.87	1.53-2.30	<0.001
Other	Ref		

*Provides recommendation and referral to screening to more than 5 patients a week.

## Discussion

This study is among the first to evaluate cancer screening practices among PHC providers in Georgia. While most providers viewed themselves as actively recommending screenings, only 21% reported recommending and referring six or more patients per week, indicating low engagement in cancer screening promotion and a perception–practice gap that may contribute to Georgia’s low national uptake (∼13%).

These findings align with global trends, indicating that limited provider-driven cancer screening engagement predominantly occurs in LMICs [10,14,19–21]. For instance, only 26% of women in Georgia learned about cervical cancer screening from healthcare providers [19]. Comparable studies in Armenia and Turkey show that only 20.6% and 38.2% of patients, respectively, received screening recommendations from family doctors, reflecting limited engagement in the region [20,22].

The intention–practice gap in Georgia likely reflects several systemic, provider, and patient-related factors resulting in underutilisation of screening programs, as seen in various settings [13,23–25]. System-level barriers included insufficient staffing, inadequate infrastructure, weak cross-level coordination, limited access to performance data, and low awareness of electronic registration tools – echoing LMIC patterns where fragmented systems impede provider-led screening [14,22]. Although screening is free nationwide, implementation quality varies by region and facility; differences in enforcement, resources, and oversight, plus provider discretion under local constraints, may affect adherence to guidance and referral activity. These organisational factors should inform the interpretation of our results. At the provider level, knowledge gaps persisted, with correct identification of screening criteria varying by cancer type. In a small Georgian study, knowledge was lowest for colorectal cancer, followed by breast and then cervical cancer [18]. Similar gaps are widely reported across LMICs [13,26]. Combined with self-reported barriers (e.g. inadequate infrastructure and limited public promotion), these findings underscore the need for targeted training and resources. Encouragingly, our multivariable analysis showed that comprehensive training was associated with a higher likelihood of recommending screening, reinforcing the value of continued education. Although differences were seen by setting (urban/rural) and role (family doctors/nurses), system- and provider-level gaps were evident across all groups. These barriers are widespread rather than role- or location-specific. Providers in Tbilisi were more likely to recommend and refer to cancer screening, suggesting urban factors may modestly facilitate provider engagement in screening promotion. Provider specialty – family doctor vs nurse – significantly influenced recommendation and referral practices. In Georgia’s PHC system, nurses are central to prevention (patient mobilisation, health education) and are expected to recommend screening and help register appointments, yet they reported lower awareness and confidence than family doctors, which may limit referral activity. Strengthening nurse engagement could therefore improve uptake. The high share of female family doctors in our sample reflects national workforce patterns. Consistent with prior studies, family doctors more often engage in preventive counselling, likely due to broader training and longer-term patient relationships, which can increase adherence to cancer screening recommendations and referral [10,27].

Perception of role was an independent predictor of behaviour: PHC providers who viewed screening as part of their job to support informed decisions were more likely to recommend and refer patients to cancer screening. This points to the value of strengthening nurses’ involvement – especially where they are the first point of contact – and fostering a shared sense of ownership for cancer prevention across all PHC staff.

### Strengths and limitations

This study offers one of the few quantitative assessments of PHC providers’ cancer-screening practices in Georgia, with a high response rate (95.3%) from a state-funded CME cohort covering over two-thirds of PHC physicians. Data collection by AFDG, a trusted agency, and a structured self-administered questionnaire adapted from the IARC tool strengthens methodological rigour and breadth.

Limitations include non-random sampling and self-report, which may misestimate behaviour. CME participants may be more motivated than the broader PHC workforce; women comprised 95% of our sample versus ∼80% nationally, potentially biasing knowledge/attitude/practice estimates. Although data were collected before the CME sessions, prior training or interest may still have influenced responses. Binary (‘yes/no’) belief items may have oversimplified nuanced views; reluctance to recommend screening can reflect evidence-based risk–benefit concerns rather than disengagement. Generalisability should therefore be interpreted with caution, and future longitudinal work should examine whether training gains translate into sustained practice change. The finding that only about 21% of providers reported referring six or more patients for cancer screening per week warrants careful interpretation. However, in Georgia – where PHC panels often exceed 2,000 patients and national programs target broad adult ages (30–70 years) – about one referral per working day (5–6/week) is a reasonable benchmark. Sensitivity analyses with alternative cut-offs (≥4, ≥8, ≥10) yielded similar predictors, suggesting that rates below six per week – particularly alongside ∼13% national uptake – more likely reflect suboptimal engagement than limited opportunity. Finally, the outcome question – ‘How many patients do you usually recommend and refer per week?’ – did not distinguish newly engaged from routinely screened patients, which may have affected responses in higher-uptake areas.

## Conclusion

This study highlights critical gaps in the cancer screening practices of PHC providers in Georgia, revealing that while there is a strong belief in the importance of cancer screening, actual referral rates are alarmingly low. The findings indicate an urgent need for comprehensive policy and systemic reforms to enhance provider education, increase awareness, and ensure adequate infrastructure that supports effective cancer screening initiatives. The study’s implications extend beyond Georgia, offering insights that may inform cancer screening practices in other low- and middle-income countries facing similar challenges.

## Supplementary Material

Supplemental Material
