# Supplementary material for: Primary healthcare providers’ knowledge, attitudes, and practices regarding cancer screening recommendation and referral in Georgia, 2023
Source: Eur J Gen Pract. 2025 Nov 24;31(1):2582292. doi: 10.1080/13814788.2025.2582292 (PMC12646092; doi:10.1080/13814788.2025.2582292)
Supplement: Supplemental Material [file IGEN_A_2582292_SM0205.zip › IGEN_2582292_suppl_data/ejgp-2025-0033-File003.docx]

**Supplementary File 2**

**Supplementary Table 1. Healthcare provider and facility characteristics stratified by work location, Georgia, 2023**

| **Variables/Provider Characteristics** | **Total** | | **Rural** | | **Urban** | | |
| --- | --- | --- | --- | --- | --- | --- | --- |
|  | **(N=2985)** | **%** | **(N=1665)** | **%** | **(N=1320)** | **%** | ***P*-value** |
| **Specialty** |  |  |  |  |  |  |  |
| Family Doctor | 1665 | 55.78 | 1052 | 52.3 | 613 | 63.0 | <0.001 |
| Nurse | 1320 | 44.22 | 960 | 47.7 | 360 | 37.0 |  |
| **Work experience in PHC*** (Mean, SD)** | 25.6 (SD 12.9) | | 25.04 (SD 12.93) | | 25.87 (SD 12.89) | | 0.786 |
| **Gender** |  | | | | | | |
| Male | 145 | 4.86 | 125 | 6.2 | 20 | 2.1 | <0.001 |
| Female | 2840 | 95.14 | 1887 | 93.8 | 953 | 97.9 |  |
| **Age** (Mean, SD) | 57.48 (SD 10.1) | |  | |  | |  |
| **PHC facility location type** |  |  |  |  |  |  |  |
| Rural | **2012** | **67.4** | ---- | ---- | ---- | ---- |  |
| Urban | **973** | **32.6** | ---- | ---- | ---- | ---- |  |
| **PHC facility location Region** |  |  |  |  |  |  |  |
| Imereti | 533 | 17.9 | 328 | 16.3 | 205 | 21.1 | <0.001 |
| Tbilisi | 371 | 12.4 | 0 | 0 | 369 | 38.0 |  |
| Samegrelo –Zemo Svaneti | 366 | 12.3 | 276 | 13.7 | 90 | 9.2 |  |
| Kakheti | 368 | 12.3 | 345 | 17.1 | 23 | 2.4 |  |
| Adjara | 293 | 9.8 | 146 | 7.3 | 147 | 15.1 |  |
| Kvemo Kartli | 256 | 8.6 | 163 | 8.1 | 93 | 9.6 |  |
| Shida Kartli | 198 | 6.6 | 178 | 8.8 | 20 | 2.1 |  |
| Samtskhe-Javakheti | 164 | 5.5 | 158 | 7.9 | 6 | 0.6 |  |
| Guria | 149 | 5.0 | 147 | 7.3 | 2 | 0.2 |  |
| Racha-Lechkhumi and Kvemo Svaneli | 146 | 4.9 | 146 | 7.3 | 0 | 0 |  |
| Mtskheta-Mtianeti | 141 | 4.7 | 123 | 6.1 | 18 | 1.8 |  |

**Supplementary Table 2. Cancer screening recommendation and referral practices of primary healthcare providers and system level factors stratified by work location, Georgia, 2023**

| **Variables/Provider Characteristics** | **Total** | | **Rural** | | **Urban** | |  |
| --- | --- | --- | --- | --- | --- | --- | --- |
|  | **(N=2985)** | **%** | **(N=2012)** | **%** | **(N=973)** | **%** | ***P*-value** |
| **Practice related to facilitating cancer screening** | | | | | | | |
| **Actively recommend and cancer screening (Yes)** | 2936 | 98.4 | 1907 | 95.2 | 931 | 95.7 | 0.117 |
| **Number of patients referred for cancer screening** |  |  |  |  |  |  |  |
| 0 | 420 | 14.1 | 420 | 14.1 | 139 | 14.3 | 0.628 |
| 1-5 | 1933 | 64.8 | 1933 | 64.8 | 610 | 62.7 |  |
| 6-10 | 426 | 14.3 | 426 | 14.3 | 148 | 15.2 |  |
| 11-15 | 95 | 3.2 | 95 | 3.2 | 37 | 3.8 |  |
| 16-20 | 45 | 1.5 | 45 | 1.5 | 15 | 1.5 |  |
| More than 20 | 66 | 2.2 | 66 | 2.2 | 24 | 2.5 |  |
| **Healthcare system-level factors to cancer screening** | | | | | | | |
| **Cancer screening program is defined by a policy framework (Yes)** | **2693** | **90.2** | 1840 | 91.5 | 853 | 87.7 | <0.001 |
| **Protocol/guideline for cancer screening (Yes)** | **2562** | **85.8** | 1765 | 87.7 | 797 | 81.9 | <0.001 |
| **Trained on cancer screening including how to advise people to get screened, benefits and risks (Yes)** | **2250** | **75.6** | 1526 | 76.1 | 724 | 74.4 | 0.3 |
| **System in place for identifying target population** (**Yes)** | **1737** | **58.2** | 1201 | 59.7 | 536 | 55.1 | 0.017 |
| **System in place for inviting eligible individuals for screening (Yes)** | **1159** | **38.8** | 787 | 39.1 | 372 | 38.2 | 0.643 |
| **System in place for notifying the results and informing about follow-up (Yes)** | **1184** | **39.7** | 359 | 36.9 | 825 | 41.0 | 0.032 |
| **System in place for sending recall notice to the non-compliant individuals (Yes)** | **696** | **23.0** | 196 | 20.1 | 200 | 24.9 | 0.004 |
| **Evaluation of performance of the programme which is published and accessible for providers (Yes)** | **446** | **14.9** | 134 | 13.8 | 312 | 15.5 | 0.213 |
| **Sufficient number of providers to ensure screening programme (Yes)** | **385** | **12.9** | 139 | 14.3 | 246 | 12.2 | 0.116 |
| **High staff turnover, which prevents proper continuity of the programme (Yes)** | **142** | **4.8** | 45 | 4.6 | 97 | 4.8 | 0.813 |
| **Adequate infrastructure for screening (Yes)** | **637** | **21.3** | 236 | 24.3 | 401 | 19.9 | 0.007 |
| **Adequate infrastructure for further management (Yes)** | **363** | **12.2** | 129 | 13.3 | 234 | 11.6 | 0.202 |
| **Long waiting lists for screening (Yes)** | **144** | **4.8** | 47 | 4.8 | 97 | 4.8 | 0.991 |
| **Long waiting lists for further management in case of screen positive result (Yes)** | **179** | **6.0** | 52 | 5.3 | 127 | 6.3 | 0.297 |
| **Adequate coordination between different healthcare levels (Yes)** | **377** | **12.6** | 114 | 11.7 | 263 | 13.1 | 0.296 |
| **Proper public promotion of the screening programme (Yes)** | **723** | **24.2** | 237 | 24.4 | 486 | 24.2 | 0.904 |
| **Cancer screening visit registration electronic system for patients (Yes)** | **792** | **26.5** | 265 | 27.2 | 527 | 26.2 | 0.545 |

**Supplementary Table 3. Cancer screening related knowledge and perceptions of primary healthcare providers stratified by work location, Georgia, 2023**

| **Variables/Provider Characteristics** | **Total** | | **Rural** | | **Urban** | |  |
| --- | --- | --- | --- | --- | --- | --- | --- |
|  | **(N=2985)** | **%** | **(N=2012)** | **%** | **(N=973)** | **%** | ***P*-value** |
| **Knowledge** | | | | | | | |
| **Knows about the national cervical cancer screening target age** | **2236** | **74.9** | 1505 | 74.8 | 731 | 75.1 | 0.847 |
| **Knows about the national breast cancer screening target age** | **1792** | **60.0** | 1219 | 60.6 | 573 | 58.9 | 0.289 |
| **Knows about the national colorectal cancer screening target age** | **1548** | **51.9** | 1040 | 51.7 | 508 | 52.2 | 0.790 |
| **Knows about the cervical cancer screening frequency** | **1408** | **47.2** | 922 | 45.8 | 486 | 49.9 | 0.034 |
| **Knows about the national breast cancer screening frequency** | **1763** | **59.1** | 1197 | 59.5 | 566 | 58.2 | 0.491 |
| **Knows about the national colorectal cancer screening frequency** | **1165** | **39.1** | 806 | 40.1 | 359 | 36.9 | 0.097 |
| **Knowledge about cancer screening program. Scale** (Mean, SD) | **3.3 (SD 1.6)** | | 3.3 **(SD 1.61)** | | 3.3 **(SD 1.47)** | | 0.064 |
| **Perceptions** | | | | | | | |
| **Believe that cancer screening is effective in decreasing cancer morbidity (Yes)** | **2955** | **98.9** | 1986 | 99.1 | 967 | 99.4 | 0.076 |
| **Believe that cancer screening is effective in decreasing cancer mortality (Yes)** | **2960** | **99.2** | 1992 | 99.4 | 968 | 99.5 | 0.138 |
| **Motivated to recommend cancer screening to patients** |  |  |  |  |  |  |  |
| Strongly Agree | **1047** | **35.1** | 632 | 31.4 | 415 | 42.7 | 0.765 |
| Somewhat agree | **1889** | **63.3** | 1346 | 66.9 | 543 | 55.8 |  |
| Other** | **49** | **1.6** | 34 | 1.7 | 15 | 1.5 |  |
| **Well informed about cancer screening procedures** |  |  |  |  |  |  |  |
| Strongly Agree | **584** | **19.6** | 215 | 22.1 | 369 | 18.3 | 0.015 |
| Somewhat agree | **2092** | **70.1** | 664 | 68.3 | 1428 | 70.9 |  |
| Other | **309** | **10.3** | 93 | 9.6 | 215 | 10.8 |  |
| **Role of primary healthcare provider in cancer screening is important** |  |  |  |  |  |  |  |
| Strongly Agree | **789** | **26.4** | 296 | 30.4 | 493 | 20.6 | <0.001 |
| Somewhat agree | **2068** | **69.3** | 615 | 63.2 | 1453 | 73.8 |  |
| Other | **128** | **4.3** | 62 | 6.4 | 66 | 5.6 |  |
| **Have enough time to discuss cancer screening program to the patients** |  |  |  |  |  |  |  |
| Strongly Agree | **401** | **13.4** | 123 | 12.6 | 278 | 13.8 | 0.377 |
| Somewhat agree | **2132** | **71.4** | 609 | 62.6 | 1523 | 75.7 |  |
| Other | **452** | **15.2** | 241 | 24.8 | 211 | 10.5 |  |
| **Cancer screening is higher priority than other healthcare issues** |  |  |  |  |  |  |  |
| Strongly Agree | **598** | **20.0** | 207 | 21.3 | 391 | 19.4 | 0.239 |
| Somewhat agree | **2160** | **72.4** | 664 | 68.2 | 1496 | 74.4 |  |
| Other | **227** | **7.6** | 102 | 10.5 | 125 | 6.2 |  |
| **Part of my job is to help people make an informed decision** |  |  |  |  |  |  |  |
| Strongly Agree | **754** | **26.1** | 491 | 25.2 | 263 | 27.8 | 0.113 |
| Somewhat agree | **2042** | **70.6** | 1397 | 71.8 | 645 | 68.3 |  |
| Other | **94** | **3.3** | 57 | 3.0 | 37 | 3.9 |  |
| **Confident that can help people make an informed decision** |  |  |  |  |  |  |  |
| Strongly Agree | **622** | **21.6** | 211 | 22.4 | 411 | 21.2 | 0.541 |
| Somewhat agree | **2137** | **74.0** | 694 | 73.5 | 1444 | 74.4 |  |
| Other | **127** | **4.4** | 39 | 4.1 | 87 | 4.4 |  |
| **Having necessary knowledge to help people make an informed screening decision** |  |  |  |  |  |  |  |
| Strongly Agree | **481** | **16.7** | 165 | 17.5 | 316 | 16.3 | 0.990 |
| Somewhat agree | **2117** | **73.4** | 687 | 72.8 | 1431 | 73.7 |  |
| Other | **286** | **9.9** | 91 | 9.7 | 194 | 10.0 |  |
