# Supplementary material for: Primary healthcare providers’ knowledge, attitudes, and practices regarding cancer screening recommendation and referral in Georgia, 2023
Source: Eur J Gen Pract. 2025 Nov 24;31(1):2582292. doi: 10.1080/13814788.2025.2582292 (PMC12646092; doi:10.1080/13814788.2025.2582292)
Supplement: Supplemental Material [file IGEN_A_2582292_SM0205.zip › IGEN_2582292_suppl_data/ejgp-2025-0033-File002.doc]

**Supplementary file 1**

**Cancer Screening Barriers: Research Questionnaire**

| **#** | **Question** | Response Options | | | | | | |
| --- | --- | --- | --- | --- | --- | --- | --- | --- |
| Healthcare Provider Characteristics | | | | | | | | |
| 1. | **Region** |  | | | | | | |
| 2. | **District** |  | | | | | | |
| 3. | **Facility location type** | □ Rural area  □ Semi-urban/village  □ Urban / City | | | | | | |
| 4. | **Position/Specialty** | □Doctor  □ Nurse  □ Clinic Manager  □ Public Health Center Employee  □ Other | | | | | | |
| 5. | **Work experience in PHC** | ------ Year | | | | | | |
| 6. | **Gender** | □ Female  □ Male | | | | | | |
| 7. | **Age** | ---- Year | | | | | | |
| 8. | **Marital status** | □ Married  □ Living with a partner  □ Never married  □ Separated  □ Divorced  □ Widowed  □ Other | | | | | | |
| 9. | **Do you have children?** | □ No  □ Yes, 1–2 children  □ Yes, 3 or more children  □ Prefer not to say | | | | | | |
| Provider-level barriers | | | | | | | | |
| 10. | Motivated to recommend cancer screening to patients | 1. Strongly disagree 2. Disagree 3. Neither agree nor disagree 4. Agree 5. Strongly agree 6. Don't know 7. Prefer not to say | | | | | | |
| 11. | **Well informed about cancer screening procedures** | 1. Strongly disagree 2. Disagree 3. Neither agree nor disagree 4. Agree 5. Strongly agree 6. Don't know 7. Prefer not to say | | | | | | |
| 12. | **Role of primary healthcare provider in cancer screening is HIGHLY important** | 1. Strongly disagree 2. Disagree 3. Neither agree nor disagree 4. Agree 5. Strongly agree 6. Don't know 7. Prefer not to say | | | | | | |
| 13. | **Have enough time to discuss cancer screening program to the patients** | 1. Strongly disagree 2. Disagree 3. Neither agree nor disagree 4. Agree 5. Strongly agree 6. Don't know 7. Prefer not to say | | | | | | |
| 14. | **Cancer screening is higher priority than other healthcare issues** | 1. Strongly disagree 2. Disagree 3. Neither agree nor disagree 4. Agree 5. Strongly agree 6. Don't know 7. Prefer not to say | | | | | | |
| Patient-level barriers | | | | | | | | |
| 15. | **What are the most common reasons your patients do not undergo cancer screening?** (Select all that apply) | □ Lack of awareness or information  □ Do not perceive cancer as a serious threat  □ Difficulty understanding information/instructions  □ Other health concerns take priority  □ Lack of trust in healthcare providers or the system.  □ Anxiety, fear, or embarrassment about the procedure  □ Anxiety or fear about diagnosis  □ Anxiety or fear about treatment.  □ Unaware that the test is free  □ Inability to follow up on positive screening results  □ Inability to afford treatment  □ Cultural or religious beliefs  □ Female patients are less likely to screen if a male conducts the test | | | | | | |
| 16. | **What are the main access-related barriers your patients face?** (Select all that apply) | □ Long distance  □ High indirect cost (e.g., travel expenses, loss of workday salary, etc.)  □ Competing priorities (e.g., caring for family members, work, etc.)  □ Physical barriers (e.g., disability)  □ Language barriers | | | | | | |
| **Healthcare system-level barriers Do the following statements correspond to the cancer screening program in Georgia?** | | | | | | | | |
| 17. | **Cancer screening program is defined by a policy framework** | □ Yes  □ No  □ I don't know | | | | | | |
| 18. | **Protocol/guideline for cancer screening** | □ Yes  □ No  □ I don't know | | | | | | |
| 19. | **Contents of the cancer screening protocol or guideline** *(Select all that apply)* | □ Description of the target population  □ Specification of the screening interval  □ Description of the screening test  □ Referral pathways and case management procedures  □ Monitoring and evaluation mechanisms  □ Regular updates to the protocol/guideline | | | | | | |
| 20. | **Trained on cancer screening including how to advise people to get screened, benefits and risks** |  Yes   No   I don't know | | | | | | |
| 21. | Availability of components within the state cancer screening program.  (Select all that apply) | □ System in place for identifying target population  □ System in place for inviting eligible individuals for screening  □ System in place for notifying the results and informing about follow-up  □ System in place for sending recall notice to the non-compliant individuals  □ Evaluation of performance of the programme which is published and accessible for providers  □ Sufficient number of providers to ensure screening programme  □ High staff turnover, which prevents proper continuity of the programme  □ Adequate infrastructure for screening  □ Adequate infrastructure for further management  □ Long waiting lists for screening  □ Long waiting lists for further management in case of screen positive result  □ Adequate coordination between different healthcare levels  □ Proper public promotion of the screening programme  □ Cancer screening visit registration electronic system for patients | | | | | | |
| Medical Staff Perspectives on Cancer Screening: Knowledge, Attitudes, and Practices | | | | | | | | |
| 22. | **Cancer screening is effective in decreasing cancer morbidity?** |  Yes   No | | | | | | |
| 23. | **For which types of cancer is screening considered effective in reducing morbidity**  (Select all that apply) |  Cervical cancer   Breast cancer   Colorectal cancer   Lung cancer   Gastric cancer   Prostate cancer   Other: ____ | | | | | | |
| 24. | Perceived effectiveness of cancer screening in reducing cancer-related mortality |  Yes   No   I don't know | | | | | | |
| 25. | **Cancer screening is effective in decreasing cancer mortality?**  (Select all that apply) |  Cervical cancer   Breast cancer   Colorectal cancer   Lung cancer   Gastric cancer   Prostate cancer   Other: ____ | | | | | | |
| 26. | **Do you actively recommend cancer screening to patients** |  Yes   No | | | | | | |
| 27. | **How many patients do you recommend and refer to cancer screening to per week**? | --- | | | | | | |
| 28. | Which types of cancer screenings do you routinely recommend? (Select all that apply) |  Cervical cancer   Breast cancer   Colorectal cancer   Lung cancer   Gastric cancer   Prostate cancer   Other: ____ | | | | | | |
| Please indicate the extent to which you agree with the following statements for each type of cancer screening listed below. | | | | | | | | |
| 29. | **Part of my job is to help people make an informed decision to be screened** | 1. Strongly disagree | 2. Disagree | 3. neither agree nor disagree | 4. Agree | 5.Completely agree | 6. Don't know | 7. prefer not to say |
|  | Cervical cancer screening |  |  |  |  |  |  |  |
|  | Breast cancer screening |  |  |  |  |  |  |  |
|  | Colorectal cancer screening |  |  |  |  |  |  |  |
| 30. | **Confident that I can help people make an informed decision about screening** | 1. Strongly disagree | 2. Disagree | 3. neither agree nor disagree | 4. Agree | 5.Completely agree | 6. Don't know | 7. prefer not to say |
|  | Cervical cancer screening |  |  |  |  |  |  |  |
|  | Breast cancer screening |  |  |  |  |  |  |  |
|  | Colorectal cancer screening |  |  |  |  |  |  |  |
| 31. | **Having necessary knowledge to help people make an informed decision to undergo cancer screening** | 1. Strongly disagree | 2. Disagree | 3. neither agree nor disagree | 4. Agree | 5.Completely agree | 6. Don't know | 7. prefer not to say |
|  | Cervical cancer screening |  |  |  |  |  |  |  |
|  | Breast cancer screening |  |  |  |  |  |  |  |
|  | Colorectal cancer screening |  |  |  |  |  |  |  |
| 32. | **Received training on providing screening advice, including communication of benefits and risks.** | 1. Strongly disagree | 2. Disagree | 3. neither agree nor disagree | 4. Agree | 5.Completely agree | 6. Don't know | 7. prefer not to say |
|  | Cervical cancer screening |  |  |  |  |  |  |  |
|  | Breast cancer screening |  |  |  |  |  |  |  |
|  | Colorectal cancer screening |  |  |  |  |  |  |  |
| Cervical Cancer Screening Utilization Among Female Healthcare Professionals (Aged 25–60) | | | | | | | | |
| 33. | Have you ever had a cervical cancer screening? |  Yes   No   I don't know | | | | | | |
| 34. | If yes, how many times have you had a cervical cancer screening in your lifetime? |  Once   Twice   Three times or more   I don't know | | | | | | |
| 35. | When was the last time you had a cervical cancer screening? |  Within the last 0–3 years  Within the last 4–5 years   A longer time ago   Never   I don't know | | | | | | |
| 36. | If you have never undergone cervical cancer screening or missed your most recent recommended screening, please indicate the reason(s). (You may select more than one) |  I didn’t know about cervical cancer screening   I don’t think the test is necessary   I thought the test would be expensive   I’m afraid of the potential costs of treatment if cancer is detected   I was worried that the cervical cancer screening might be painful   My family members didn’t support me   Fear of a bad result   Negative experience with cervical cancer screening in the past   I’m not sure the test is done safely   Feeling of shame   I didn’t want the test to be performed by a male doctor   The test is unacceptable from a cultural/religious perspective   It wasn’t possible to schedule an appointment at a convenient time for me   I am too busy/it’s inconvenient   I have had a hysterectomy   I’ve received treatment for gynecological cancer   I was not yet 25 years old   I don’t know/it’s not relevant | | | | | | |
| 37. | Have you ever been vaccinated against the human papillomavirus (HPV) |  Yes   No   I don't know | | | | | | |
| 38. | If applicable, do you intend to undergo cervical cancer screening? | (1) Definitely not (2) Probably not (3) I find it hard to decide (4) Probably yes (5) Definitely yes | | | | | | |
| 39. | I am confident that, if I wanted to, I would know where and how to undergo a cervical cancer screening | (1) Definitely not (2) Probably not (3) I find it hard to decide (4) Probably yes (5) Definitely yes | | | | | | |
| 40. | I believe that cervical cancer screening is important for protecting my health | (1) Definitely not (2) Probably not (3) I find it hard to decide (4) Probably yes (5) Definitely yes | | | | | | |
| 41. | I believe cervical cancer can be extremely dangerous | (1) Definitely not (2) Probably not (3) I find it hard to decide (4) Probably yes (5) Definitely yes | | | | | | |
| 42. | The following questions relate to your views and experiences with cervical cancer screening. |  My husband or partner believes I should regularly participate in cervical cancer screening.   My family believes I should regularly participate in cervical cancer screening.   I have sufficient information to make an informed decision about participating in cervical cancer screening.   In general, people in Georgia support cervical cancer screening.   believe that cervical cancer screening is shameful or embarrassing.   A healthcare professional has recommended that I participate in cervical cancer screening.   I know other women who have undergone cervical cancer screening. | | | | | | |
